# HLA Polymorphisms and COVID‐19 Susceptibility and Severity: Insights From an Iranian Patients Cohort

**DOI:** 10.1111/jcmm.70570

**Published:** 2025-05-14

**Authors:** Pooria Hakimi, Kasra Arbabi Zaboli, Mohammadreza Golbabapour‐Samakoush, Susan Azizimohammadi, Fatemeh Soleimani, Mohammad Hosein Salmani, Ladan Teimoori‐Toolabi

**Affiliations:** ^1^ Molecular Medicine Department, Biotechnology Research Center Pasteur Institute of Iran Tehran Iran; ^2^ Hajar Hospital Tehran Iran

**Keywords:** COVID‐19, HLA‐A antigens, HLA‐B antigens, HLA‐C antigens, HLA‐DR antigens

## Abstract

The HLA system is a crucial immune response component against infectious agents, including SARS‐CoV‐2. Certain polymorphisms may impart varying levels of protection or vulnerability to COVID‐19. This research aims to understand the possible relationship between HLA polymorphisms and the susceptibility to COVID‐19 and its severity. We recruited 290 hospitalised Iranian COVID‐19 patients (130 severe and 160 moderate). Using PCR‐SSP methods, we conducted a detailed analysis of polymorphisms in HLA class I (HLA‐A, HLA‐B, and HLA‐C) and II (HLA‐DRB1 and HLA‐DQB1) molecules at low resolution. The study found that certain HLA alleles, including HLA‐B*49, HLA‐B*52, HLA‐C*12, HLA‐DRB1*04, and HLA‐DQB1*05, were associated with disease susceptibility. Additionally, HLA‐A*23, DRB1*10, and DRB1*13 were indicators of disease severity. The study also noted that individuals carrying the HLA‐A*23 allele showed a significant decrease in lymphocyte levels and an elevated likelihood of developing thrombosis. We hypothesise that a maladaptive immune response may occur based on these findings. This might be due to the strong affinity of the HLA‐A*23 allele group for presenting a wide range of SARS‐CoV‐2 peptides. Such a presentation possibly leads to a cytokine storm, followed by lymphocyte apoptosis and an increase in platelet count.

## 1 Introduction

COVID‐19, or Coronavirus Disease 2019, is an acute respiratory infection caused by an RNA virus called Severe Acute Respiratory Syndrome Coronavirus 2 (SARS‐CoV‐2). The severity of this illness varies, ranging from no symptoms to pneumonia and potentially life‐threatening outcomes [1]. Research has shown a clear link between COVID‐19 susceptibility and certain health comorbidities, such as advanced age, hypertension, obesity, diabetes, heart disease, and respiratory illnesses [2, 3]. However, it is important to identify and understand the factors that may significantly impact the immune response and to differentiate the roles of these factors in the susceptibility and severity of COVID‐19.

The human leukocyte antigens (HLA) are important proteins involved in the adaptive immune response. The HLA gene locus is located within the 6p21.3 region, and it is the most polymorphic area in the human genome. HLAs present pathogen‐derived peptides on the surface of infected cells, helping to activate specific T lymphocytes and leading to an appropriate immune response against the pathogen [4, 5, 6]. The HLA locus exhibits high diversity rates, with thousands of documented polymorphisms. These genetic variations could lead to individual differences in immune responses to pathogens [7]. Furthermore, the combination of HLA alleles (haplotypes) diversifies the effectiveness of the immune response to different pathogens. The selective pressure exerted by the pathogens throughout evolution helps survival of these haplotypes. Variances in COVID‐19 susceptibility and mortality among patients from different regions of the world may be partly due to an uneven distribution of HLA alleles and haplotypes [8, 9, 10].

Studies have looked into the significance of different HLA alleles in predicting individual outcomes of SARS‐CoV‐2 infection [11, 12, 13]. For instance, in an observational study, Correale et al. identified HLA‐C*01 and B*44 alleles as potential genetic factors linked to the spread of COVID‐19 in Italy [11]. However, there has been limited research on this correlation in the Iranian population [14, 15, 16]. In a study by Ebrahimi et al., the distribution of HLA class II alleles was examined in 144 COVID‐19 patients split into three groups based on clinical manifestation. They observed a reverse relationship between the presence of the DRB1*04 allele group and COVID‐19 severity (OR = 0.289, *p* = 0.005). However, they cautioned against over‐interpreting their findings, mentioning the study's limitations, such as small sample size and a lack of investigation into HLA class I alleles. Other studies have also investigated the connection between HLA alleles and SARS‐CoV‐1, a virus closely related to SARS‐CoV‐2. They discovered that patients with the HLA‐B*46:01 allele are more likely to experience severe symptoms, while those with the HLA‐B*07:03 and HLA‐DRB1*03:01 alleles are more susceptible to the disease [17].

The potential importance of HLA signatures in determining COVID‐19 prognosis led us to believe that these may play a crucial role in the outcome of disease. To test this hypothesis, we performed HLA typing at low resolution using Polymerase Chain sequence‐specific Primers (PCR‐SSP) in 290 Iranian hospitalised patients with SARS‐CoV‐2 infection. Our goal was to investigate any potential correlation between HLA class I (HLA‐A, HLA‐B, HLA‐C) and class II (HLA‐DR and HLA‐DQ) allele groups with the disease susceptibility, severity, as well as other clinical and laboratory data.

## 2 Results

### 2.1 Clinical Characteristics Among Two Subgroups of the COVID‐19 Patients

Table 1 compares the baseline clinical characteristics between two subgroups of patients, revealing significant differences in all variables between moderate and severe COVID‐19 patients. Notably, several signs were significantly associated with severe COVID‐19, including increased Respiratory Rate (RR) (*p* < 0.001) and decreased oxygen saturation (*p* < 0.001). The severe group had lower mean lymphocyte counts (*p* < 0.001) and elevated mean serum levels of aspartate aminotransferase (AST) (*p* < 0.001) and mean alanine aminotransferase (ALT) (*p* < 0.001). The mean levels of C‐reactive protein (CRP), an inflammatory marker, were significantly higher in the severe group (*p* < 0.001). Additionally, the Length of Hospital Stay was significantly longer in severe COVID‐19 cases (*p* < 0.001).

**TABLE 1:** Clinical characteristics among two subgroups of COVID‐19 patients based on disease severity.

Clinical variable	Patients' subgroup		*p*
	Moderate (*n* = 160)	Severe (*n* = 130)	
Age (years) (mean ± SD, range)	49.77 ± 16.83 (18–79)	55.21 ± 13.26 (21–80)	0.013
Gender (male/female)	72/88 (45.3%–54.7%)	55/75 (42.1%–57.9%)	0.672
Mortality rate (%)	0.0	23.0	< 0.001
Length of hospital stay (days)	4.87 ± 2.50 (1–17)	9.43 ± 6.40 (2–39)	< 0.001
Mean PaO_2_ (%)	92.66 ± 2.029 (90–98)	81.32 ± 9.879 (35–91)	< 0.001
Mean volume of oxygen (Lit)	2.68 ± 4.220 (0–10)	10.24 ± 3.735 (2–15)	< 0.001
Maximum WBC (*n*/μL)	8285.91 ± 3236.866 (2800–18,900)	12471.52 ± 5743.519 (1100–35,800)	< 0.001
Mean CRP (mg/Lit)	35.86 ± 32.573 (1–196)	72.89 ± 45.200 (3–201)	< 0.001
Maximum RR (breaths/min)	20.66 ± 6.166 (14–28)	24.65 ± 9.022 (22–40)	< 0.001
Minimum LYM (*n*/μL)	1199.28 ± 575.031 (264–3355)	732.04 ± 327.799 (198–1728)	< 0.001
Mean LYM (*n*/μL)	1430.63 ± 566.99 (432–3376.5)	1084.74 ± 421.42 (403.25–2913)	< 0.001
Mean PLT (*n*/μL) *1000	263.53 ± 89.881 (114–647)	323.81 ± 114.770 (80–690)	< 0.001
Mean PR (pulse/min)	89.02 ± 12.225 (55–143)	99.61 ± 16.531 (73–160)	< 0.001
Mean ANC (*n*/μL)	6384.10 ± 3147.124 (1240–17,112)	11091.92 ± 5315.528 (2812–34,010)	< 0.001
Mean Alk/P (U/Lit)	210.01 ± 82.815 (46–666)	266.04 ± 147.949 (75–958)	< 0.001
Mean Hb (g/Lit)	13.67 ± 1.493 (10–17)	13.24 ± 1.731 (8–17)	0.148
Mean FBS (mg/dL)	180.82 ± 57.802 (80–350)	151.57 ± 75.415 (88–256)	0.002
Mean AST (U/L)	46.62 ± 31.017 (2–238)	71.27 ± 58.649 (13–486)	< 0.001
Mean ESR (mm/h)	39.78 ± 21.191 (2–97)	51.67 ± 22.223 (7–112)	< 0.001
Mean ALT (U/L)	39.78 ± 53.545 (10–443)	56.43 ± 91.121 (11–591)	< 0.001

Abbreviations: ALT, alanine transaminase; Alk/P, alkaline phosphatase; ANC, absolute neutrophil count; AST, aspartate transaminase; CRP, C‐reactive protein; ESR, erythrocyte sedimentation rate; FBS, fasting blood sugar; HB, haemoglobin; LOS, length of stay; LYM, lymphocyte; PLT, platelet (thrombocyte) count; PO_2_, partial pressure of oxygen; PR, pulse rate; RR, respiration rate; WBC, white blood cell.

**FIGURE 1:**
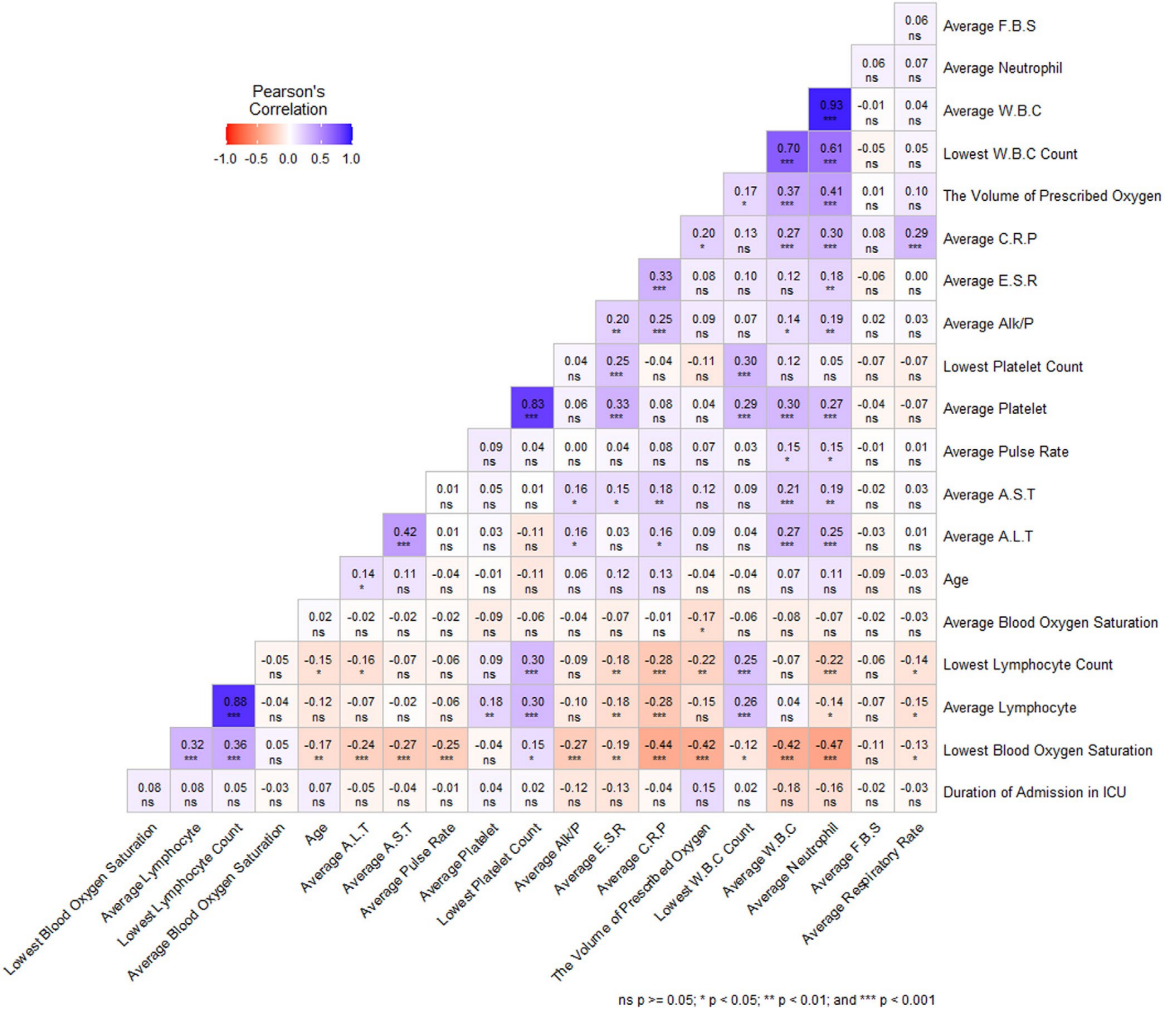
Heatmap illustrating the correlation between clinical variables in COVID‐19 patients. Each cell represents the Pearson correlation coefficient, with positive values indicating a direct correlation between variables and negative values reflecting an inverse relationship. The correlations were represented using colours; the red spectrum indicates a negative correlation, while the purple spectrum represents a positive correlation. The intensity of colours corresponds to the strength of correlation.

Furthermore, Figure 1 includes a heat map illustrating the correlation analysis between the variables. The map shows Pearson correlation scores and two‐tailed significant P‐values. The analysis reveals interconnections between several clinical manifestations. For example, the average white blood cell (WBC) count, along with the average neutrophil count, has a negative correlation with the lowest blood oxygen saturation levels and a positive correlation with average levels of ALT, AST, Alk/P, platelets, and CRP. Moreover, the average lymphocyte level shows a positive correlation with average platelet and average ESR levels, while displaying a negative correlation with the average levels of CRP.

### 2.2 HLA Class I/II Profile May Affect COVID‐19 Susceptibility

In this study, we aimed to investigate the relationship between HLA classes I/II allele groups and the prevalence of COVID‐19. To accomplish this objective, we compared the frequencies of HLA alleles in a cohort of 290 COVID‐19 patients and pooled prevalence estimates for various human leukocyte antigen types in Iran (Table 2). We also examined whether there were any deviations from Hardy–Weinberg equilibrium (HWE) for all the HLA loci in the entire patient cohort, as detailed in Data S1. The analysis showed no significant departures from HWE.

**TABLE 2:** HLA class I/II allele‐groups comparison between COVID‐19 patients (*n* = 290) and combined prevalence estimates for multiple HLA types.

HLA allele‐groups	Patients' frequency, (2n = 580)	Reference population (%)/Overall *I* ^2^ (%)/Egger's test (*p*)	OR (95% CI)	*p*	Pc
A*23	4.1 (24)	2.4/93.01/0.03	1.71 (0.974–3.030)	0.059	NS
A*66	0.6 (4)	0.1/82.74/0.05	7.315 (0.759–70.495)	0.077	NS
A*12	0.1 (1)	2.0/96.48/0.74	0.098 (0.130–0.713)	0.002	**0.03**
A*28	0.1 (1)	8.1/97.51/0.23	0.022 (0.003–0.172)	< 0.001	**0.011**
B*49	4.8 (28)	1.8/81.26/0.78	2.83 (1.564–5.153)	< 0.001	**0.02**
B*52	6.7 (39)	3.1/77.63/0.17	2.250 (1.407–3.597)	< 0.001	**0.015**
B*44	5.3 (31)	3.1/67.65/0.16	1.76 (1.690–2.901)	0.037	NS
B*51	14.3 (83)	10.8/90.57/0.17	1.384 (1.022–1.875)	0.035	NS
B*35	19.8 (115)	13.8/90.95/0.02	1.53 (1.174–2.010)	0.002	**0.04**
B*18	6.3 (37)	3.11/97.87/0.71	2.109 (1.309–3.396)	0.003	NS
B*13	5.5 (32)	3.1/82.90/0.61	1.83 (1.116–3.000)	0.019	NS
C*05	1.5 (9)	0.1/78.03/0.08	19.910 (2.318–171.112)	0.002	**0.024**
C*04	17.2 (100)	10.0/98.09/0.01	1.885 (1.327–2.678)	< 0.001	**0.01**
C*12	21.9 (127)	15.0/89.51/0.88	1.589 (1.161–2.174)	0.004	**0.045**
C*16	5.9 (34)	3.3/36.51/0.91	1.813 (1.024–3.208)	0.039	NS
C*01	3.6 (21)	10.8/99.35/0.006	0.307 (0.164–0.570)	< 0.001	**0.01**
C*02	3.6 (21)	10.0/99.31/0.001	0.336 (0.179–0.631)	< 0.001	**0.01**
C*18	0.3 (2)	5.0/90.0/0.38	0.062 (0.034–1.064)	< 0.001	**0.01**
DRB1*04	9.1 (53)	4.8/83.90/0.39	2.009 (1.316–3.068)	0.001	**0.012**
DQB1*05	29.1 (169)	20.6/77.24/0.47	1.573 (1.183–2.092)	0.002	**0.01**
DQB1*03	7.2 (42)	10.5/92.38/0.73	0.665 (0.515–0.889)	0.045	NS
DRB1*03	28.7 (167)	37.3/85.76/0.02	0.677 (0.445–0.993)	0.005	NS

*Note:* Corrected *p*‐values that are statistically significant are highlighted in bold format.

**FIGURE 2:**
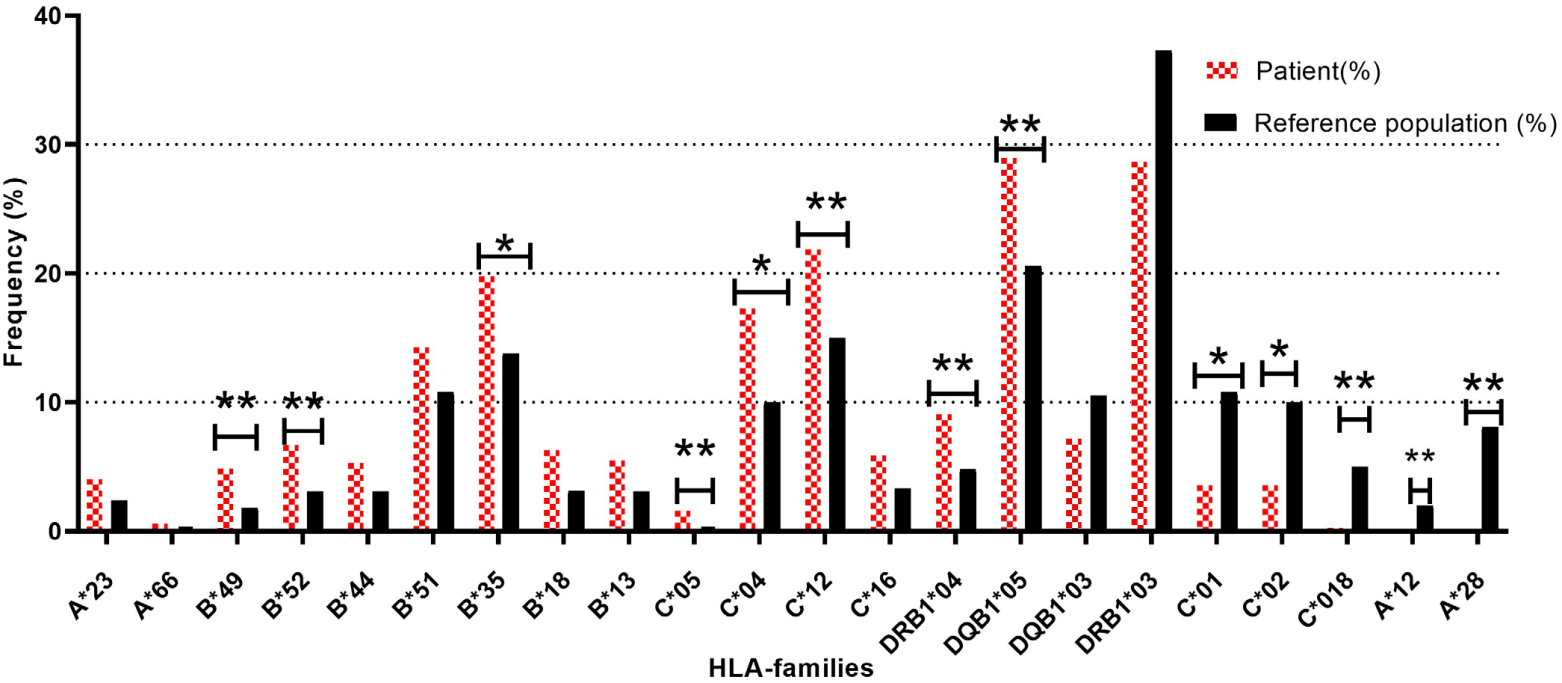
Prevalence of HLA class I/II profiles in COVID‐19 patients and reference population: Indications of significance marked with an asterisk (*) for HLA allele groups with considerable publication bias and/or heterogeneity of studies in the reference population. More conservative differences are highlighted by two asterisks (**).

We discovered that the patient group had higher frequencies of certain HLA alleles, including HLA‐B*49 (OR = 2.83, corrected *p*‐value (Pc) = 0.02), HLA‐B*52 (OR = 2.250, Pc = 0.015), HLA‐B*35 (OR = 1.53, Pc = 0.04), HLA‐C*05 (OR = 1.53, Pc = 0.04), HLA‐C*04 (OR = 1.885, Pc = 0.01), HLA‐C*12 (OR = 1.589, Pc = 0.045), HLA‐DRB1*04 (OR = 2.009, Pc = 0.012), and HLA‐DQB1*05 (OR = 1.573, Pc = 0.01). In contrast, we observed lower frequencies of HLA‐A*12 (OR = 0.098, Pc = 0.03), HLA‐A*28 (OR = 0.022, Pc = 0.011), HLA‐C*01 (OR = 0.307, Pc = 0.01), HLA‐C*02 (OR = 0.336, Pc = 0.01), and HLA‐C*18 (OR = 0.062, Pc = 0.01) after adjusting the P‐values using the Bonferroni correction in the patient group (Figure 2).

#### 2.2.1 HLA Allele Groups, Genotype, Haplotype, and Homozygosity Frequency Comparison Among COVID‐19 Patient Subpopulation

We analysed and compared the occurrence rates of various HLA allele groups, genotypes, and haplotypes within severe (*n* = 130) and moderate classes (*n* = 160). The genotype frequencies of the HLA‐A, HLA‐B, HLA‐C, HLA‐DRB1, and HLA‐DQB1 loci were found to be in the Hardy–Weinberg equilibrium, except for HLA‐A and HLA‐C in severe cases (*p*‐value = 0.002 and < 0.001, respectively) and HLA‐B in the moderate group (*p*‐value < 0.001). These findings are presented in Data S2.

**FIGURE 3:**
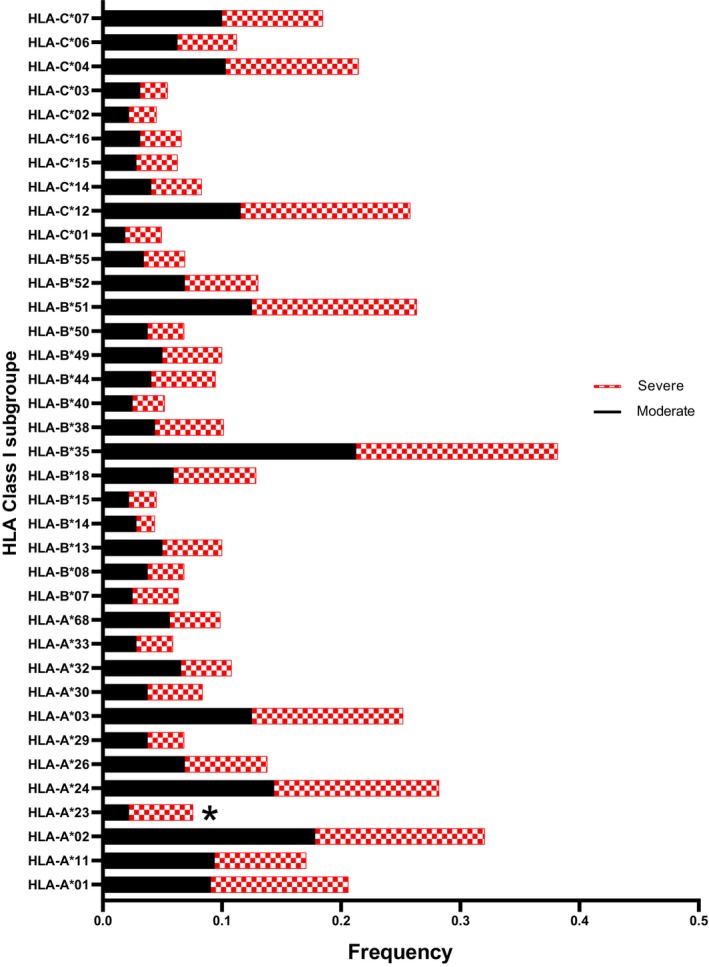
Comparison of HLA Class I allele group frequencies between subpopulations of COVID‐19 patients (severe vs. moderate). Significance indicated by asterisk (*).

**FIGURE 4:**
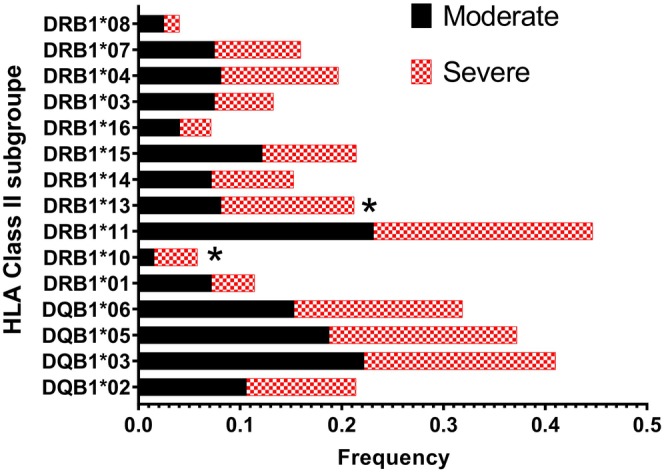
Comparison of HLA Class II allele group frequencies among subpopulations of COVID‐19 patients (severe vs. moderate). Significant levels are indicated by an asterisk (*).

Table 3 gives a summary of the distribution of HLA‐allele groups. These results were generated using the BIGDAWGE package and are visually represented in Figures 3 and 4. Importantly, the frequency of HLA‐A*23 (OR = 2.63, Pc = 0.033), HLA‐DRB1*10 (OR = 2.74, Pc = 0.044), and HLA‐DRB1*13 (OR = 1.68, Pc = 0.048) allele groups showed a higher prevalence in severe cases after adjusting the *p*‐values using the Bonferroni correction.

**TABLE 3:** HLA allele group frequency comparison among COVID‐19 patient subpopulation.

HLA allele–groups	Frequency in patients subgroups		OR (95% CI)	*p*	Pc
	Moderate group frequency (2n = 320)	Severe group frequency (2n = 260)			
HLA‐A*01	0.0906 (29)	0.1153 (30)	1.36 (0.76–2.42)	0.26865	NS
HLA‐A*11	0.0937 (30)	0.0769 (20)	0.83 (0.44–1.56)	0.54193	NS
HLA‐A*02	0.1781 (57)	0.1423 (37)	0.79 (0.49–1.27)	0.31168	NS
HLA‐A*23	0.0218 (8)	0.0538 (16)	2.63 (0.97–7.81)	0.003057	**0.033**
HLA‐A*24	0.1437 (46)	0.1384 (36)	0.99 (0.6–1.63)	0.97108	NS
HLA‐A*26	0.0687 (22)	0.0692 (18)	1.04 (0.51–2.09)	0.90382	NS
HLA‐A*29	0.0375 (12)	0.0307 (8)	0.84 (0.29–2.28)	0.70779	NS
HLA‐A*03	0.1250 (40)	0.1269 (33)	1.05 (0.62–1.78)	0.83629	NS
HLA‐A*30	0.0375 (12)	0.0461 (12)	1.28 (0.52–3.18)	0.55083	NS
HLA‐A*32	0.0656 (21)	0.0423 (11)	0.65 (0.28–1.44)	0.25445	NS
HLA‐A*33	0.0281 (9)	0.0307 (8)	1.13 (0.37–3.36)	0.80203	NS
HLA‐A*68	0.0562 (18)	0.0423 (11)	0.76 (0.32–1.75)	0.49293	NS
HLA‐B*07	0.0250 (9)	0.0384 (10)	1.63 (0.57–4.82)	0.30848	NS
HLA‐B*08	0.0375 (13)	0.0307 (8)	0.85 (0.3–2.3)	0.72439	NS
HLA‐B*13	0.0500 (18)	0.0500 (14)	1.04 (0.45–2.37)	0.91237	NS
HLA‐B*14	0.0281 (10)	0.0153 (4)	0.56 (0.13–2.05)	0.33645	NS
HLA‐B*15	0.0218 (7)	0.0230 (6)	1.1 (0.3–3.88)	0.86493	NS
HLA‐B*18	0.0593 (21)	0.0692 (16)	1.23 (0.59–2.54)	0.54171	NS
HLA‐B*35	0.2125 (73)	0.1692 (42)	0.79 (0.5–1.23)	0.27654	NS
HLA‐B*38	0.0437 (14)	0.0538 (13)	1.4 (0.61–3.19)	0.3794	NS
HLA‐B*40	0.0250 (9)	0.0269 (7)	1.12 (0.34–3.6)	0.82303	NS
HLA‐B*44	0.0406 (16)	0.0538 (15)	1.4 (0.6–3.31)	0.38965	NS
HLA‐B*49	0.0500 (16)	0.0538 (12)	1.04 (0.45–2.37)	0.91237	NS
HLA‐B*50	0.0375 (13)	0.0307 (8)	0.85 (0.3–2.3)	0.72439	NS
HLA‐B*51	0.1250 (45)	0.1384 (38)	1.18 (0.7–1.97)	0.50447	NS
HLA‐B*52	0.0687 (23)	0.0615 (16)	0.93 (0.44–1.9)	0.82321	NS
HLA‐B*55	0.0343 (11)	0.0346 (9)	1.05 (0.38–2.84)	0.91523	NS
HLA‐C*01	0.0287 (10)	0.0307 (8)	1.64 (0.48–5.85)	0.36779	NS
HLA‐C*12	0.1496 (45)	0.1423 (37)	1.26 (0.73–2.19)	0.37065	NS
HLA‐C*14	0.0406 (16)	0.0423 (11)	1.02 (0.4–2.55)	0.96494	NS
HLA‐C*15	0.0381 (12)	0.0346 (9)	1.21 (0.42–3.55)	0.68744	NS
HLA‐C*16	0.0412 (13)	0.0346 (9)	1.09 (0.38–3.06)	0.85948	NS
HLA‐C*02	0.0218 (11)	0.0230 (6)	1.03 (0.28–3.67)	0.95576	NS
HLA‐C*03	0.0412 (13)	0.0230 (6)	0.71 (0.21–2.22)	0.5157	NS
HLA‐C*04	0.1031 (40)	0.1115 (29)	1.07 (0.59–1.92)	0.81085	NS
HLA‐C*06	0.0625 (30)	0.0500 (13)	0.76 (0.34–1.68)	0.46621	NS
HLA‐C*07	0.1450 (43)	0.0846 (22)	0.8 (0.42–1.5)	0.45413	NS
HLA‐DQB1*02	0.1559 (51)	0.1609 (42)	1.04 (0.58–1.86)	0.8783	NS
HLA‐DQB1*03	0.3256 (106)	0.2816 (73)	0.82 (0.52–1.29)	0.36249	NS
HLA‐DQB1*05	0.2752 (90)	0.2758 (72)	1.01 (0.63–1.61)	0.96676	NS
HLA‐DQB1*06	0.2247 (73)	0.2471 (64)	1.14 (0.69–1.87)	0.58661	NS
HLA‐DRB1*01	0.0793 (25)	0.0458 (12)	0.56 (0.24–1.22)	0.11742	NS
HLA‐DRB1*10	0.0156 (5)	0.0458 (12)	2.74 (0.86–10.18)	0.0044	**0.044**
HLA‐DRB1*11	0.2551 (82)	0.2333 (61)	0.89 (0.58–1.35)	0.56079	NS
HLA‐DRB1*13	0.0896 (29)	0.1416 (37)	1.68 (0.94–3.01)	0.0048	**0.048**
HLA‐DRB1*14	0.0793 (25)	0.0875 (23)	1.11 (0.57–2.17)	0.73375	NS
HLA‐DRB1*15	0.1344 (43)	0.1000 (26)	0.72 (0.4–1.26)	0.22209	NS
HLA‐DRB1*16	0.0448 (14)	0.0333 (9)	0.73 (0.26–1.95)	0.49953	NS
HLA‐DRB1*03	0.0827 (26)	0.0625 (16)	0.74 (0.35–1.51)	0.37392	NS
HLA‐DRB1*04	0.0896 (29)	0.1250 (33)	1.45 (0.8–2.64)	0.18764	NS
HLA‐DRB1*07	0.0827 (26)	0.0916 (24)	1.12 (0.58–2.15)	0.71691	NS
HLA‐DRB1*08	0.0275 (9)	0.0166 (4)	0.6 (0.13–2.27)	0.40024	NS

*Note:* Corrected P‐values that are statistically significant are highlighted in bold format.

**FIGURE 5:**
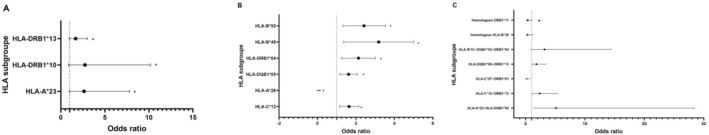
(A) Risk estimates of HLA allele groups significantly associated with disease severity in subpopulations of COVID‐19 patients. (B) Risk estimates of homozygote and haplotype HLA allele groups related to disease severity. (C) Risk estimates of homozygote and haplotype HLA allele groups related to disease severity. Significant levels are indicated by an asterisk (*).

The higher frequency levels observed for HLA‐A*23, HLA‐DRB1*10, and HLA‐DRB1*13 suggest a potential association between the presence of these allele groups and the severity of COVID‐19 infection. There was no significant difference in genotype distribution among the participants based on the analysis conducted (Data S3). Figure 5 illustrates the risk estimates of HLA allele groups that exhibit significant differences in patient subgroups and serve as the representatives of disease severity.

In Table 4, we see the frequencies of potential haplotypes and their corresponding *p*‐values, which are approaching significance concerning the severity of the disease. The distribution of the following haplotypes exhibited significant differences among the moderate and severe subpopulations: A*23 ~ DQB1*03 (OR = 5.11, *p*‐value = 0.005), C*12 ~ DRB1*13 (OR = 2.37, *p*‐value = 0.016), C*07 ~ DRB1*01 (OR = 0.21, *p*‐value = 0.006), DQB1*06 ~ DRB1*13 (OR = 1.83, *p*‐value = 0.035), and B*51 ~ DQB1*03 ~ DRB1*04 (OR = 3.2, *p*‐value = 0.044). The data indicate that certain genetic combinations (haplotypes) A*23 ~ DQB1*03, C*12 ~ DRB1*13, DQB1*06 ~ DRB1*13, and B*51 ~ DQB1*03 ~ DRB1*04 were more common in patients with severe COVID‐19, while the C*07 ~ DRB1*01 haplotype was more prevalent in patients with a moderate form of the disease compared to those with severe symptoms. All possible haplotypes including two, three, four, and five possible haplotypes were analysed, but since no significant levels of difference were observed between our groups, they are not mentioned. The distribution of all the observed haplotypes can be found in Data S4.

**TABLE 4:** Differences in the haplotype frequencies between moderate and severe COVID‐19 subgroups with approaching significant *p*‐values.

Haplotype	Frequency in patients' subgroups		OR (95% CI)	*p*	Pc
	Moderate (2n = 320)	Severe (2n = 260)			
A*23 ~ DQB1*03	0.0093 (3)	0.0461 (12)	5.11 (1.36–28.44)	**0.005516**	NS
A*32 ~ DQB1*05	0.0500 (16)	0.0230 (6)	0.45 (0.14–1.23)	0.091412	NS
A*02 ~ DRB1*11	0.0750 (24)	0.0384 (10)	0.49 (0.21–1.1)	0.062476	NS
A*03 ~ DRB1*11	0.0312 (10)	0.0615 (16)	2.03 (0.85–5.1)	0.079582	NS
B*35 ~ DRB1*14	0.0218 (7)	0.0307 (8)	1.42 (0.44–4.66)	0.50213	NS
B*51 ~ DRB1*13	0.0156 (5)	0.0384 (10)	2.52 (0.77–9.51)	0.084852	NS
C*12 ~ DRB1*13	0.0375 (12)	0.0846 (22)	2.37 (1.1–5.37)	**0.016298**	NS
C*12 ~ DRB1*14	0.0375 (12)	0.0730 (19)	2.02 (0.91–4.66)	0.058169	NS
C*12 ~ DRB1*15	0.0906 (29)	0.0538 (14)	0.57 (0.27–1.15)	0.092699	NS
C*07 ~ DRB1*01	0.0531 (17)	0.0115 (3)	0.21 (0.04–0.73)	**0.006339**	NS
DQB1*05 ~ DRB1*01	0.0656 (21)	0.0307 (8)	0.45 (0.17–1.09)	0.055432	NS
DQB1*05 ~ DRB1*10	0.0156 (5)	0.0423 (11)	2.78 (0.88–10.34)	0.051031	NS
DQB1*06 ~ DRB1*13	0.0687 (22)	0.1192 (31)	1.83 (1–3.42)	**0.035883**	NS
B*51 ~ DQB1*03 ~ DRB1*04	0.0187 (6)	0.0535 (14)	3.2 (0.87–14.42)	**0.044907**	NS

*Note:* *p*‐values that are statistically significant are highlighted in bold format.

The presence of homozygote HLA‐B*35 (OR = 0.21, *p*‐value = 0.042) and HLA‐DRB1*11 (OR = 0.35, *p*‐value = 0.009) in patients with a moderate form of COVID‐19 was significantly higher compared to those with a severe form of the disease. However, it is important to note that the statistical significance for HLA‐B*35 did not remain significant after applying the Bonferroni correction, so it should be interpreted with caution.

#### 2.2.2 Exploring the Correlations Between HLA Allele Groups, Haplotypes, and Clinical Manifestations: A Statistical Analysis

Certain HLA genetic variations were associated with specific changes in blood cell counts and other clinical markers. Table 6 presents the results of Levene's test for equality of variances and t‐tests for equality of means related to important clinical manifestations that are associated with specific HLA allele groups (Figure S1) or haplotypes (Figure S2). In the group of patients being studied, individuals with the HLA‐B*49, HLA‐A*23, and A*23 ~ DQB1*03 showed a significantly higher average difference in their maximum platelet count (Maximum PLT) compared to non‐carriers (*p*‐value = 0.023, *p*‐value = 0.020, and *p*‐value = 0.001 respectively). For instance, individuals with HLA‐DRB1*10 and B*51 ~ DQB1*03 ~ DRB1*04 showed a significant decrease in their lowest observed lymphocyte count (*p*‐value = 0.050 and 0.02 respectively). On the other hand, those carrying HLA‐DRB1*13 had a significantly higher mean WBC count (*p*‐value = 0.028) and lower mean PaO2 levels (*p*‐value = 0.018). Interestingly, the clinical manifestations of HLA‐A*23 revealed a notable decrease in average lymphocyte counts and an increase in the maximum platelet counts. These effects were more prominent among individuals carrying both HLA‐A*23 and DQB1*03, as indicated in Table 6. Furthermore, individuals with C*07 ~ DRB1*01 had significantly lower levels of WBC and neutrophil count (*p*‐value = 0.001 and 0.03, respectively). Additionally, patients with a possible DQB1*06 ~ DRB1*13 haplotype exhibited higher levels of WBC (*p*‐value = 0.05).

**FIGURE 6:**
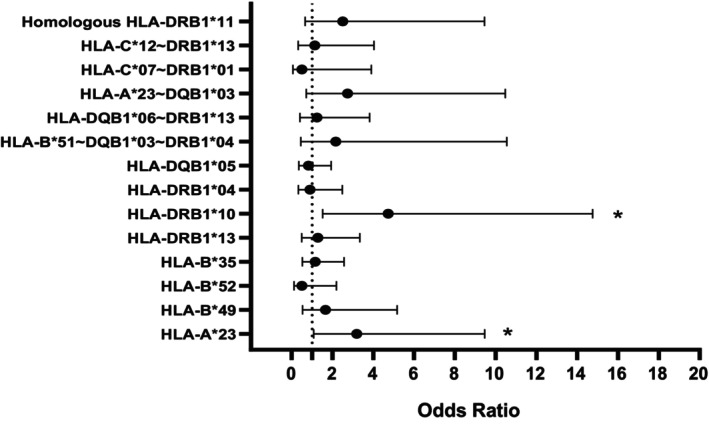
HLA allele groups risk estimates related to mortality rates. Significant levels are denoted by an asterisk (*).

It is noted that HLA‐A*23 and HLA‐DRB1*10 may have a significant impact on mortality in COVID‐19 patients (Table 7). The mortality odds ratio for HLA‐A*23 carriers is 3.19 with a *p*‐value of 0.045. Similarly, for HLA‐DRB1*10 carriers, the odds ratio is 4.73 with a *p*‐value of 0.014 (Figure 6). These results highlight the possible role of HLA genetic factors in determining susceptibility to severe COVID‐19.

## 3 Discussion

The role of HLA genotypes in determining the severity of COVID‐19 has been investigated in various regions. However, there is limited information about this association in the Middle East, especially in Iran. This study aimed to examine the connection between HLA genotypes and COVID‐19 severity in 290 hospitalised COVID‐19 patients, making it one of the largest patient cohorts in Iran to undergo HLA typing. The study focused on Iranian patients diagnosed with moderate to severe COVID‐19. The results indicated that specific HLA alleles were significantly associated with severe COVID‐19 pneumonia, while other allele groups provided protection. HLA molecules play crucial roles in various aspects of the immune response during viral infections. Different HLA genotypes may lead to diverse T‐cell immune responses, potentially impacting the symptoms and outcome of the disease [18, 19].

The majority of viral peptides presented by the MHC‐I molecules on infected cells have the potential to induce an immune response [20]. Therefore, it could be advantageous to have HLA molecules with high binding capabilities for viral peptides from novel viral infections, like SARS‐CoV‐2, on the surface of antigen‐presenting cells [21]. The enhanced binding affinity of HLA molecules for viral peptides may be associated with a more robust immune response in patients carrying these specific HLA alleles or haplotypes, leading to better outcomes. However, it is important to consider that in certain situations, the opposite may also be true, as proposed by Gutiérrez‐Bautista and colleagues. They suggested that an effective display of SARS‐CoV‐2 antigens could boost the cytotoxic CD8+ T cell response, causing greater damage to lung tissue and an increased risk of death in severe COVID‐19 cases. Moreover, it could also stimulate the activation of T helper lymphocytes (Th), further exacerbating the inflammatory state [22].

In this study, we aimed to investigate the relationship between HLA alleles and susceptibility to SARS‐CoV‐2 infection as well as the associated clinical signs and symptoms. To do this, we compared the distribution of these alleles between hospitalised COVID‐19 patients and healthy controls. Furthermore, we categorised the COVID‐19 patients into moderate and severe subgroups based on their clinical presentation and analysed their HLA allele groups, genotypes, haplotypes, and homozygosity to identify any potential association with disease severity. Our analysis revealed specific groups of HLA alleles that may be linked to an increased susceptibility to SARS‐CoV‐2 infection (Table 2). Several studies have reported conflicting findings regarding the association between some of these HLA alleles and COVID‐19 outcomes.

Our research indicates that the HLA‐B*49 allele group is linked to disease susceptibility. In Russia, Bubnova et al. reported that the HLA‐B*49 allele group is associated with an increased susceptibility to infection with the Delta (B.1.617.2) strain of COVID‐19 [23]. However, a contrasting finding by Correale et al. suggests that HLA‐B*49 is a negative predictor for COVID‐19 incidence in Italy [11]. It is important to note that different populations may have distinct genetic and environmental factors influencing COVID‐19 susceptibility. Additionally, a study in Japan demonstrated a significant association between HLA‐B*52:01:01:02 and the severity of COVID‐19, supporting our findings about the role of HLA‐B*52 in COVID‐19 susceptibility [24].

Per our research on the impact of the HLA‐C*12 allele group on susceptibility to disease, a study carried out in Japan involving 137 patients with mild COVID‐19 (mCOVID‐19) and 53 patients with severe COVID‐19 provided evidence suggesting that HLA‐C*12:02:02:01 could be a dependable indicator of disease severity [24].

It has been noted in our investigation that HLA‐DQB1*05 could be an indicator of susceptibility to the disease. This was supported by a study conducted by Gutiérrez‐Bautista et al., which found a higher frequency of HLA‐DQB1*05:03 in COVID‐19 patients requiring hospitalisation compared to the control group. However, it is important to note that the observed significance level did not remain statistically significant after correcting for multiple comparisons of P‐values [24]. Another observation of our study was the higher prevalence of HLA‐B*35 among COVID‐19 patients. In connection with this, Leite and colleagues conducted a study to explore the relationship between the worldwide frequencies of HLA‐B alleles and the daily death rates (DDR) resulting from COVID‐19 [25]. Their study identified HLA‐B*35:01 and HLA‐B*35:03 as harmful alleles, which is in line with our observation of a significant increase in HLA‐B*35 frequency among hospitalised COVID‐19 patients [25]. Importantly, many peptides from the SARS‐CoV‐2 proteome showed strong binding affinity to the HLA‐B35*:01 and HLA‐B35*:03 alleles, with 191 and 147 peptides, respectively. A study conducted in Iran examined the prevalence of HLA‐A, HLA‐B, HLA‐C, and HLA‐DRB1 at the two‐digit resolution level in 142 COVID‐19 patients and 143 healthy individuals from the same ethnic background. A comparison of allele frequencies between the two groups revealed potential predisposing effects of HLA‐A*03, B*35, and DRB1*16 alleles [26].

Numerous studies have indicated that HLA‐C*04 may influence the course of COVID‐19 infections [27, 28, 29, 30]. Researchers, including Weiner and colleagues, found that individuals with the HLA‐C*04:01 allele are more likely to experience a severe clinical course of SARS‐CoV‐2 infection. This conclusion is supported by data from four countries: Germany, Spain, the United States, and Switzerland, as well as independent genome‐wide association studies. The researchers believe their findings are biologically plausible, given that HLA‐C*04:01 has fewer predicted binding sites for the relevant SARS‐CoV‐2 peptides than other HLA alleles [29]. Additionally, combining multiple *in silico* HLA predictors identified C*04:01 as a potentially significant risk factor for COVID‐19 [28]. The observed higher prevalence of HLA‐C*04 in COVID‐19 patients is supported by all the above‐mentioned reports.

In COVID‐19 patients, we observed a notable increase in the occurrence of HLA‐DRB1*04. This finding aligns with a prior study by Hajebi et al. The study, which used a low‐resolution HLA typing test on a relatively small sample size (15 COVID‐19 patients and 10 healthy controls), reported a higher occurrence of HLA‐DRB1*04 in severe COVID‐19 participants in Iran [15]. Recent research has also suggested that HLA‐DRB1*04 could potentially serve as a predictor of COVID‐19 severity [16, 31, 32]. Langton et al. conducted a thorough analysis of classical HLA genes, including class I and class II, in 147 individuals of European descent with varying clinical outcomes after COVID‐19 infection. Using next‐generation sequencing (NGS), the authors reported a significant difference in the frequency of the HLA‐DRB1*04:01 allele between severe COVID‐19 patients and asymptomatic participants (5.1% vs. 16.7%). This provides further evidence for the potential role of HLA in COVID‐19 pathogenesis. Interestingly, a separate study in Iran found no significant difference in the prevalence of HLA‐DRB1*04 between COVID‐19 patients and healthy controls. They also reported significantly lower frequencies of HLA‐DRB1*04 in the moderate subgroup in comparison with the severe group, though it did not remain significant after correction for multiple tests [17]. Furthermore, Wang et al. genotyped 82 Chinese COVID‐19 patients for HLA‐A, ‐B, ‐C, ‐DRB1, ‐DRB3/4/5, ‐DQA1, ‐DQB1, ‐DPA1, and ‐DPB1 loci using NGS [33]. They found higher levels of HLA‐DRB1*04:06 in the patient group compared to healthy controls (7.32% vs. 3.20%). However, after Bonferroni correction, this difference lost its significance [33]. The difference in the prevalence of HLA‐DRB1*04 in our study compared to previous research may be due to variations in the resolution at which HLA‐DRB1*04 alleles were examined. It is important to note that HLA‐DRB1*04 encompasses a range of HLA alleles, including HLA‐DRB1*04:01 and HLA‐DRB1*04:06, among others. Therefore, when analysed as a group, these different HLA subtypes within HLA‐DRB1*04 may have compensatory effects.

In our study, we found evidence suggesting that certain HLA alleles, including HLA‐A*12, HLA‐A*28, HLA‐C*01, HLA‐C*02, and HLA‐C*18, may protect against SARS‐CoV‐2 infection (Table 2). However, we refrained from drawing definitive conclusions due to significant variations in the prevalence of HLA‐C*01 and HLA‐C*02 among COVID‐19 patients compared to the reference group. This variation was attributed to substantial heterogeneity and publication bias in the frequency of the reference group (as indicated in Table 2). The substantial heterogeneity, reflected in the I^2^ statistic, implies notable differences in the prevalence of these specific HLA alleles within the studies involved in the systematic review from which our reference group was obtained. In other words, the individuals in the reference group had diverse genetic profiles concerning these particular alleles. The study looked at the connection between HLA gene polymorphisms and the severity of COVID‐19. It found that HLA‐A*23, HLA‐DRB1*10, and HLA‐DRB1*13 were more prevalent among patients with severe disease (Table 3). Littera and his team investigated the impact of HLA class I and II molecules on susceptibility to SARS‐CoV‐2 infection in the Sardinian population, prompted by the historically low infection rate in this group [27]. Their findings showed a significant increase in the frequency of HLA‐A*23:01 among patients with moderate or severe COVID‐19 compared to those with mild symptoms or no symptoms. However, a separate *in silico* analysis suggested that HLA‐A*23:01 might be protective because it can present a larger quantity of SARS‐CoV‐2 peptides [34].

Our findings related to HLA‐DRB1*10 revealed an odds ratio of 2.74 and a corrected *p*‐value of 0.044 in association with disease severity, consistent with findings from previous studies. Additionally, we noted a significant increase in mortality rates among individuals with HLA‐DRB1*10, providing further evidence of its association with both COVID‐19 susceptibility and severity of COVID‐19. Ebrahimi and colleagues, in a cohort of 144 individuals, reported lower frequencies of the HLA‐DRB1*10 allele group in critically and severely ill Iranian patients with COVID‐19 compared to those with moderate illness [16]. However, this observation lost statistical significance after correction for multiple comparisons, suggesting it may have been due to chance or other confounding factors. In contrast, Poulton and colleagues reported a higher prevalence of HLA‐DRB1*10 in individuals with severe clinical symptoms [31].

In line with our research, which showed a higher prevalence of HLA‐DRB1*13 allele groups in severe cases, a study on a Saudi Arabian population also found that DRB1*13 was significantly more common in patients with the severe and fatal form of the disease compared to those with mild infections. However, the statistical significance of the association between DRB1*13 and the severity of COVID‐19, as well as all other alleles, was lost after correcting for multiple tests [35].

We have discovered several haplotypes that were significant and strongly linked with severe COVID‐19 outcomes. These include A*23 ~ DQB1*03, C*12 ~ DRB1*13, DQB1*06 ~ DRB1*13, and B*51 ~ DQB1*03 ~ DRB1*04. On the other hand, the haplotype C*07 ~ DRB1*01 was less common among severe patients. However, it is important to note that none of these associations remained statistically significant after correcting for multiple tests using Bonferroni correction.

Although HLA diversity can enhance the likelihood of survival within a population during an epidemic, our analysis of HLA allele homozygosity suggests that carrying two copies of the HLA‐DRB1*11 allele group may be associated with a reduced risk of severe COVID‐19, as presented in Table 5. A recent meta‐analysis study investigating the association between HLA‐DRB1 alleles and ICU admission has revealed an important finding. It was discovered that the HLA‐DRB1*11 allele group provides protective effects against COVID‐19‐related hospitalisation and the progression of the disease necessitating treatment in the ICU [36]. Furthermore, another meta‐analysis study showed that HLA‐DRB1*11 could be related to a decrease in the severity of COVID‐19, which could lead to intensive care unit requirements [37]. Moreover, in a study conducted in Burkina Faso, the HLA‐DRB1*11 allele was stated as a protective factor against developing COVID‐19 symptoms but not against infection with the disease [38]. Additionally, HLA‐DRB1*11:01 and HLA‐DRB1*11:02 were proposed as effective presenters of COVID‐19 peptides [39, 40].

**TABLE 5:** Homozygosity of HLA allele groups and its association with disease severity.

Homozygote HLA allele–groups	Frequency in patients' subgroups		OR (95% CI)	*p*	Pc
	Moderate (observed *n*–total *n*)	Severe (observed *n*–total *n*)			
A*21	0.28 (8–28)	0.41 (14–34)	1.75 (0.54–5.93)	0.30	NS
A*03	0.28 (8–28)	0.11 (4–34)	0.33 (0.07–1.47)	0.09	NS
B*35	0.66 (8–12)	0.30 (6–20)	0.21 (0.03–1.24)	**0.04**	NS
C*12	0.12 (4–32)	0.30 (12–40)	3.00 (0.77–14.13)	0.07	NS
C*04	0.18 (6–32)	0.20 (8–40)	1.08 (0.29–4.30)	0.89	NS
C*07	0.31 (10–32)	0.35 (14–40)	1.18 (0.40–3.62)	0.73	NS
DRB1*11	0.64 (18–28)	0.38 (14–36)	0.35 (0.11–1.10)	**0.009**	**0.043**

*Note:* Statistically significant *p*‐values are highlighted in bold format.

An analysis was conducted to identify potential connections between genetic variations in the HLA genes and the clinical presentations. It was found that individuals carrying HLA‐B*51, HLA‐ DQB1*03, HLA‐ DRB1*04, HLA‐A*23, and HLA‐A*23~DQB1*03 had significantly lower mean lymphocyte levels. Patients with HLA‐DRB1*10 and HLA‐A*23~DQB1*03 showed decreased lowest lymphocyte count. These findings provide light on the potential impact of altered HLA allele groups and haplotypes on the clinical manifestations of COVID‐19. Recent studies have also reported a high binding affinity of HLA‐A*23:01, HLA‐DQB1*03:01, and HLA‐DQB1*03:02 to SARS‐CoV‐2 epitopes [34, 41]. Pretti and colleagues have reported that A*23:01 seems to be a specialised allele for the Spike genomic regions. It can present a higher number of peptides from this region compared to the nucleocapsid genomic region. In addition, another study suggested HLA‐A*23:04 to be a strong binder to SARS‐CoV‐2 peptides, with over 100 peptides bound with high affinity [40]. These findings may provide insight into the harmful effects of these genomic variations on the progression of COVID‐19. Specifically, the over‐presentation of SARS‐CoV‐2 antigens through these HLA alleles might enhance the cytotoxic CD8+ T cell response, leading to excessive production of inflammatory markers, such as Interleukin‐6 and Interleukin‐8. This overexpression would result in a cytokine storm and consequently lead to apoptosis of lymphocytes [42]. On the other hand, Poulton et al. suggested that HLA‐DRB1*10 may have a reduced ability to present the viral peptides required for the development of a protective T‐cell repertoire. Recent studies have highlighted the significance of the C‐reactive protein to lymphocyte ratio as a predictive factor for poor clinical outcomes in patients with SARS‐CoV‐2 infection [43, 44]. We found that the average lymphocyte count had a negative correlation with average CRP levels. Also, the average white blood cell (WBC) and neutrophil count showed a positive correlation with average levels of CRP.

In our study, we found that patients with HLA‐B*49, HLA‐A*23, and HLA‐A*23 ~ DQB1*03 had higher platelet levels (Table 6). Litvinov and his team's research have revealed a significant correlation between thrombophilia and inflammation in COVID‐19 patients [45]. The laboratory results demonstrate that hypercoagulability is associated with excessive production of inflammatory markers, including Interleukin‐6 and Interleukin‐8, fibrinogen, and CRP, along with increased levels of inflammatory cells in neutrophilia and monocytosis. These findings support the established concept that a cytokine storm related to inflammation can lead to immune‐related thrombosis through various pathways [46, 47, 48]. For instance, Interleukin‐6 has been shown to trigger the expression of tissue factors in monocytes, macrophages, and endothelial cells, whereas TNF‐α and interleukin‐1 suppress the activity of natural anticoagulants [49].

**TABLE 6:** Statistical analysis of HLA allele groups and haplotypes clinical manifestations.

	*t*‐test for equality of means						
	*t*	df	*p* (2‐tailed)	Mean difference	Std. Error difference	95% confidence interval of the difference	
						Lower	Upper
HLA‐B*49							
Maximum PLT	2.285	508	**0.023**	48.696	21.307	6.836	90.557
Mean ESR	2.342	217	**0.020**	9.95	4.25	1.58	18.31
HLA‐A*23							
Mean WBC	3.118	265	**0.002**	2456.03	787.71	905.06	4007.00
Maximum PLT	2.325	492	**0.020**	54.637	23.495	8.473	100.800
Mean LYM	−1.913	269	**0.05**	−255.92	133.80	−519.36	7.51
Mean NEU	2.758	16.857	**0.014**	3277.01	1188.35	768.19	5785.84
HLA‐DRB1*10							
Lowest PO_2_	−1.82	14.70	**0.09**	−6.84	3.75	−14.85	1.17
Lowest lymphocyte count	−2.06	262.00	**0.04**	−276.65	134.42	−541.33	−11.96
HLA‐DRB1*13							
Mean WBC	2.213	265	**0.028**	1167.72	527.69	128.73	2206.71
Mean PaO_2_	−2.385	219.495	**0.018**	−35.55	14.90	−64.92	−6.18
B*51 ~ DQB1*03 ~ DRB1*04							
Mean LYM	−2.71	12.79	**0.02**	−274.40	101.33	−493.69	−55.12
DQB1*06 ~ DRB1*13							
Mean WBC	1.97	265.00	**0.05**	1225.73	623.22	−1.37	2452.83
A*23 ~ DQB1*03							
Mean WBC	1.94	265.00	**0.05**	1799.66	927.85	−27.23	3626.56
Mean LYM	−2.38	269.00	**0.02**	−369.54	155.35	−675.39	−63.69
Maximum PLT	3.423	259	**0.001**	97.171	28.386	41.274	153.068
Lowest lymphocyte count	−2.57	262.00	**0.01**	−381.77	148.73	−674.63	−88.90
Mean NEU	2.48	262.00	**0.01**	2346.72	947.47	481.09	4212.34
C*07 ~ DRB1*01							
Mean WBC	−4.29	23.23	**0.001**	−2028.03	472.80	−3005.56	−1050.51
Mean NEU	−2.19	261.00	**0.03**	−1819.96	830.12	−3454.54	−185.38

*Note:* *p*‐values that are statistically significant are highlighted in bold format.

In addition to the aforementioned observations, we have observed an interesting link between the improved mean value of absolute neutrophil count and the presence of HLA‐A*23 and HLA‐A*23 ~ DQB1*03. It is important to note that previously reported findings have highlighted significant pathophysiological changes in severe COVID‐19 cases, including marked alterations in the abundance, phenotype, and functionality of neutrophils. More specifically, following infection with SARS‐CoV‐2, there is a notable increase in the number of neutrophils present in the nasopharyngeal epithelium [50], which then spreads to other parts of the lung. COVID‐19 patients often show higher levels of neutrophils in their blood [1, 50, 51], especially in severe cases. Neutrophils can also lead to the formation of neutrophil extracellular traps (NETs), release of cytokines like Interleukin −8 (CXCL8), and recruiting other immune cells to regulate processes such as acute and chronic inflammation, which may ultimately cause Acute Respiratory Distress Syndrome (ARDS) [52].

The possible synergistic effects observed in individuals carrying the HLA‐A*23 ~ DQB1*03 haplotype are noteworthy. According to Table 6, carriers of HLA‐A*23 alone show a mean difference of + 54.6 × 10^3^/μL in their maximum platelet count (Max PLT) and − 255.9 cells/μL in mean lymphocyte levels. However, among those with the HLA‐A*23 ~ DQB1*03 haplotype, these alterations appear more pronounced, with a + 97.1 × 10^3^/μL change in Max PLT and −369 cells/μL for lymphocytes. This suggests a possible synergistic effect of both HLA‐A*23 and HLA‐DQB1*03 in influencing platelet counts and lymphocyte levels, emphasising the importance of examining specific allele combinations when investigating disease manifestations. Finally, the impact of HLA loci on the mortality of COVID‐19 patients is a crucial area of study in current medical research. Analysing carriers and non‐carriers of HLA loci has provided interesting insights into this field (Table 7). The statistical analysis of mortality rates in carriers and non‐carriers indicates that individuals with HLA‐A*23 and HLA‐DRB1*10 have significantly higher fatality rates after infection with COVID‐19. The odds ratio of mortality in HLA‐A*23 carriers (3.19) and HLA‐DRB1*10 (4.73) shows a robust correlation with this fatal outcome. These findings might have significant implications for the medical community and underscore the need for further research to understand the mechanisms linking HLA loci to mortality in COVID‐19 patients. Identifying HLA loci associated with increased mortality may help identify individuals at higher risk of severe COVID‐19 and guide the development of personalised treatment approaches.

**TABLE 7:** Impact of HLA loci on mortality: statistical comparison of carriers and non‐carriers.

Locus	Carriers		Non‐carries		OR (95% CI)	*p*
	Dead (%)	Survived (%)	Dead (%)	Survived (%)		
HLA‐A*23	23.8	76.2	8.9	91.1	3.190 (1.075–9.471)	**0.045**
HLA‐B*49	14.8	85.2	9.5	90.5	1.656 (0.530–5.171)	0.328
HLA‐B*52	5.4	94.6	10.2	89.8	0.499 (0.113–2.195)	0.551
HLA‐B*35	10.5	89.5	9.1	90.9	1.156 (0.520–2.572)	0.836
HLA‐DRB1*13	11.5	88.5	9.2	90.8	1.281 (0.492–3.335)	0.607
HLA‐DRB1*10	31.3	68.8	8.7	91.3	4.735 (1.519–14.762)	**0.014**
HLA‐DRB1*04	8.9	91.1	9.8	90.2	0.899 (0.326–2.480)	1.000
HLA‐DQB1*05	8.5	91.5	10.2	89.8	0.819 (0.347–1.934)	0.832
HLA‐B*51 ~ DQB1*03 ~ DRB1*04	18.2	81.8	9.3	90.7	2.162 (0.443–10.545)	0.288
HLA‐DQB1*06 ~ DRB1*13	11.4	88.6	9.4	90.6	1.242 (0.404–3.818)	0.759
HLA‐A*23 ~ DQB1*03	21.4	78.6	9.0	90.0	2.738 (0.716–10.469)	0.142
HLA‐C*07 ~ DRB1*01	5.3	94.7	9.9	90.1	0.502 (0.064–3.910)	1.000
HLA‐C*12 ~ DRB1*13	10.7	89.3	9.5	90.5	1.138 (0.321–4.037)	0.741
Homozygote HLA‐DRB1*11	20.0	80.0	9.0	90.0	2.500 (0.661–9.455)	0.167

*Note:* *p*‐values that are statistically significant are highlighted in bold format.

## 4 Conclusion

This study examined HLA class I and class II alleles in 290 Iranian COVID‐19 patients, who were divided into two subgroups based on their clinical characteristics. Specific HLA alleles, such as HLA‐B*49, HLA‐B*52, HLA‐C*12, HLA‐DRB1*04, and HLA–DQB1*05, were identified as indicators of disease susceptibility. Furthermore, HLA‐A*23, HLA‐DRB1*10, and HLA‐DRB1*13 were found to be possible predictors of disease severity. Based on previous research and the clinical manifestations associated with the HLA‐A*23 allele group, our investigation suggests the hypothesis that the possible high binding affinity of HLA‐A*23 antigens to diverse peptides of SARS‐CoV‐2 might trigger an exaggerated immune response upon CD8+ lymphocyte activation. As a result, this likely leads to a cytokine storm characterised by increased inflammation and subsequent lymphocyte apoptosis, ultimately resulting in lymphocytopenia. The maladaptive immune response may also increase the risk of thrombosis, which could exacerbate the severity of the disease. However, experimental validations are required to confirm this hypothesis. Conversely, the limited ability of HLA‐DRB1*10 to present viral peptides may result in an ineffective immune response, potentially leading to disease progression and worsening.

These findings emphasise the possible role of genetic factors in determining COVID‐19 susceptibility and severity. They also suggest the possibility of developing personalised treatment approaches based on HLA genotypes in the future. However, since the study utilised 2‐digit HLA typing, the results should be interpreted with caution. Future research employing high‐resolution HLA typing is necessary to confirm these potential findings.

## 5 Methods and Materials

### 5.1 Study Subjects and Settings

This study enrolled 500 COVID‐19 patients who were hospitalised at Hajar Hospital in Tehran, Iran. The diagnosis of COVID‐19 was confirmed by analysing the upper respiratory specimens (nasopharyngeal swabs) using real‐time polymerase chain reaction (RT‐PCR) for SARS‐CoV‐2. The study protocol and any amendments to it, were approved by the Ethics Committee of the National Institute of Medical Research Development (NIMAD) (*IR.NIMAD.REC.1399.088*), and all patients provided informed consent following the NIMAD and Pasteur Institute of Iran guidelines. After applying exclusion criteria that included chronic illnesses such as diabetes, renal disease, liver disease, hypertension, cardiovascular disease, and malignancies, as well as a history of other infectious diseases such as HIV and HBV, we identified 290 eligible participants for inclusion in the study. Patients were categorised as having either severe or moderate disease [53]. Severe disease was defined as the presence of respiratory distress syndrome with a Respiratory Rate (RR) ≥ 30/min, resting blood oxygen saturation ≤ 93%, or partial pressure of arterial blood oxygen (PaO2)/oxygen concentration (FiO2) ≤ 300 mmHg. This study classified patients who were hospitalised in the Intensive Care Unit (ICU) due to severe conditions requiring mechanical ventilation, organ failure, or shock as severely ill. HLA frequencies were compared with a reference population obtained from a meta‐analysis, study conducted in Iran [54], which included studies on clinically healthy participants within Iranian populations. The research assessed the statistical heterogeneity of the incorporated studies using Higgins' I^2^ statistic, which was then represented as a percentage. Additionally, Egger's test P‐values less than 0.1 were considered indicative of significant publication bias in this comprehensive investigation.

### 5.2 Preparation of Samples and Assessment of HLA Class I and II Genotypes

In this study, we obtained blood samples from patients and  collected them in EDTA K3 Vacuum Blood Collection Tubes. We extracted DNA from peripheral blood using the salting‐out method [55] and then measured its quality and quantity using a BioTek Microplate Spectrophotometer from Agilent Technologies, USA. To determine the HLA‐A, HLA‐B, HLA‐C, HLA‐DRB1, and HLA‐DQB1 loci, we used the low‐resolution Olerup SSP combi trays and HISTO TYPE SSP kits (CareDx and BAG Diagnostics, respectively) relied on the PCR‐SSP method. After PCR cycling, the entire PCR products were electrophoresed in a 2% agarose gel floated in 0.5X TBE buffer and viewed under a UV trans‐illuminator. To analyse the results obtained from Olerup SSP combi trays and HISTO TYPE SSP kits, we used SCORE and Histomatch software tools respectively. Finally, 2‐digit HLA allele groups were assigned for further analysis.

### 5.3 Clinical Characteristics and Treatment Interventions in COVID‐19 Patients

The medical team diligently recorded the clinical features and laboratory data of all patients using their medical records, including variables such as age, gender, Length of Stay (LOS) in the Hospital, and a range of parameters like White blood cell (WBC) count, C‐reactive protein (CRP), Respiration rate (RR), Lymphocyte (LYM) and Platelet (PLT‐thrombocyte) count, Pulse rate (PR), ANC or Absolute Neutrophil Count (NEU), Alkaline phosphatase (Alk/P), Haemoglobin (Hb), Fasting Blood Sugar (FBS), Aspartate transaminase (AST), Erythrocyte Sedimentation Rate (ESR) and Alanine transaminase (ALT). The term “lowest lymphocyte count” (LLC) refers to the lowest count of lymphocytes observed in patients during their hospital stay. Individualised treatments were given based on each patient's specific needs, including antiviral drugs like Remdesivir or Favipiravir and Interferon beta‐1a; anti‐coagulant drugs like heparin or enoxaparin; anti‐inflammatory agents such as dexamethasone; and additional therapy such as vitamin C, Zinc, and nonsteroidal anti‐inflammatory drugs (NSAIDs).

### 5.4 Statistical Analysis

The study compared the frequency distribution of HLA class I and II in both the case and control groups using either the chi‐square test with Yates' correction or Fisher's exact test. Statistical analysis of alleles, genotypes, and haplotypes between severe and moderate groups was conducted using the R package BIGDAWG [56] from the R Foundation for Statistical Computing. The Hardy–Weinberg Equilibrium (HWE) assumptions of our HLA data for patient groups were assessed using the GAP package (https://cran.r‐project.org/web/packages/gap/index.html). The strength of association between HLA allele groups, haplotypes, genotypes, and homozygosity was studied using the odds ratio (OR) with a 95% confidence interval. To address the multiplicity of testing, significance levels were adjusted by Bonferroni correction. A corrected P value of less than 0.05 was considered statistically significant for all tests. Quantitative data was analysed using independent sample t‐tests and Welch's t‐test. Bivariate Pearson Correlation was employed by the Multi‐Environment Trial Analysis (metan) package (https://cran.r‐project.org/web/packages/metan/index.html) in R to analyse and illustrate the correlation of clinical data. The Overall *I* ^2^ (%) and Egger's test (*p*‐value) are essential for assessing the heterogeneity and publication bias within the meta‐analysis. The overall *I* ^2^ (%) measures the variability across studies due to heterogeneity rather than chance. Egger's test (p‐value) helps evaluate potential small‐study effects or bias. These statistical analyses were performed utilising SPSS V.27.0.1.0. Representative figures were created using GraphPad Prism V.8.4.3.

## Author Contributions

**Pooria Hakimi:** conceptualization (supporting), data curation (lead), formal analysis (lead), methodology (supporting), validation (supporting), writing – original draft (supporting). **Kasra Arbabi Zaboli:** data curation (lead), formal analysis (lead), methodology (lead), software (lead), validation (lead), writing – original draft (lead). **Mohammadreza Golbabapour‐Samakoush:** formal analysis (supporting), investigation (supporting). **Susan Azizimohammadi:** investigation (supporting), resources (supporting). **Fatemeh Soleimani:** software (supporting), validation (supporting). **Mohammad Hosein Salmani:** data curation (supporting), formal analysis (supporting). **Ladan Teimoori‐Toolabi:** conceptualization (lead), funding acquisition (lead), project administration (lead), resources (lead), supervision (lead), writing – review and editing (lead).

## Ethics Statement

The study protocol and amendments were approved by the Ethics Committee of the National Institute of Medical Research Development (NIMAD) (IR.NIMAD.REC.1399.088). All patients have signed the informed consent.

## Conflicts of Interest

The authors declare no conflicts of interest.

## Supporting information


Figure S1.

Data S1.

Data S2.

Data S3.

Data S4.

## Data Availability

Data associated with our study has been deposited into a publicly available repository (data base.rar).
