# Supplementary material for: HLA Polymorphisms and COVID‐19 Susceptibility and Severity: Insights From an Iranian Patients Cohort
Source: J Cell Mol Med. 2025 May 14;29(9):e70570. doi: 10.1111/jcmm.70570 (PMC12077277; doi:10.1111/jcmm.70570)
Supplement: Supplementary file 1 — Figure S1. [file JCMM-29-e70570-s003.docx]

Supplementary figures


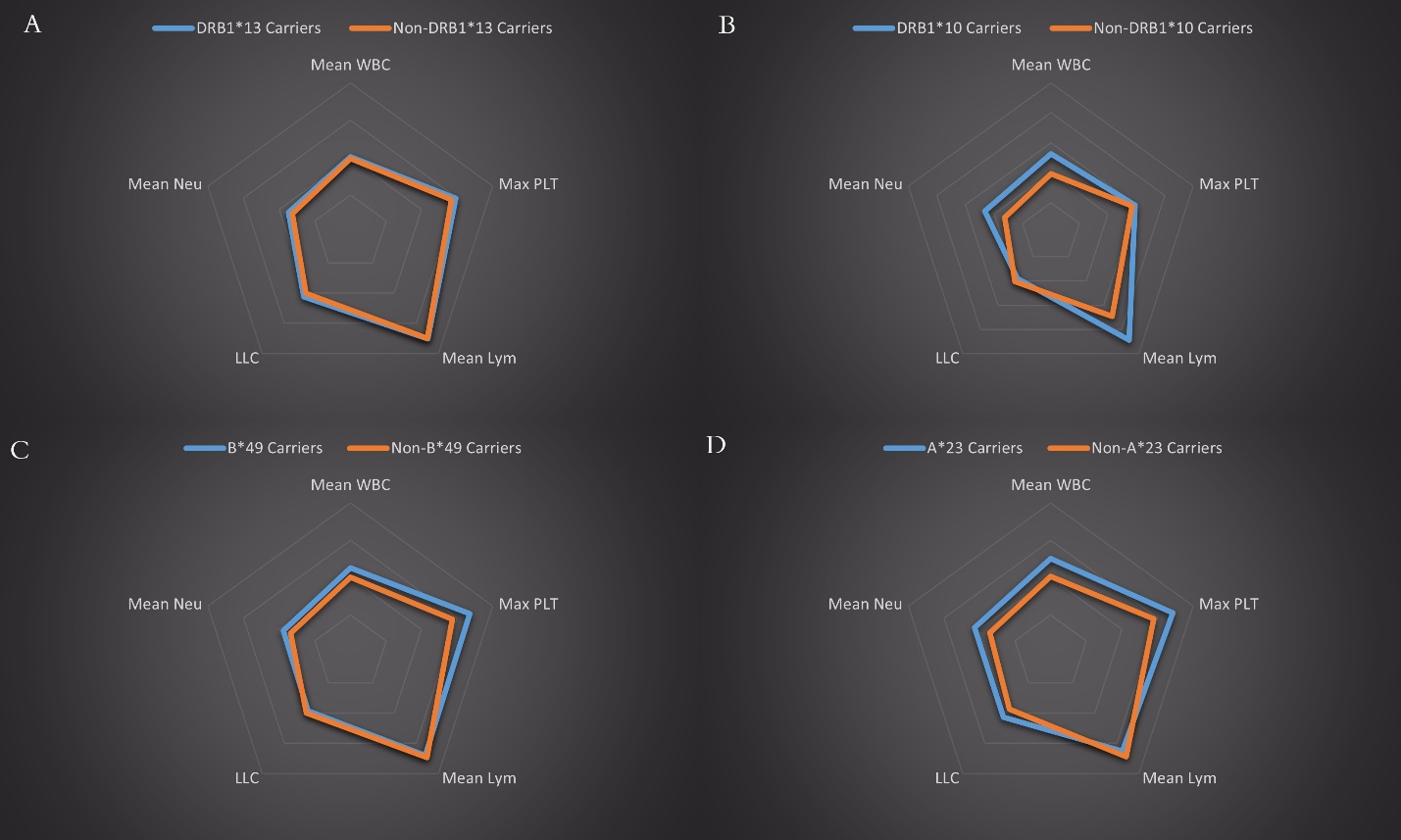


**Figure S1**. Radar chart illustrating clinically significant differences between individuals carrying and those not carrying specific HLA allele groups.


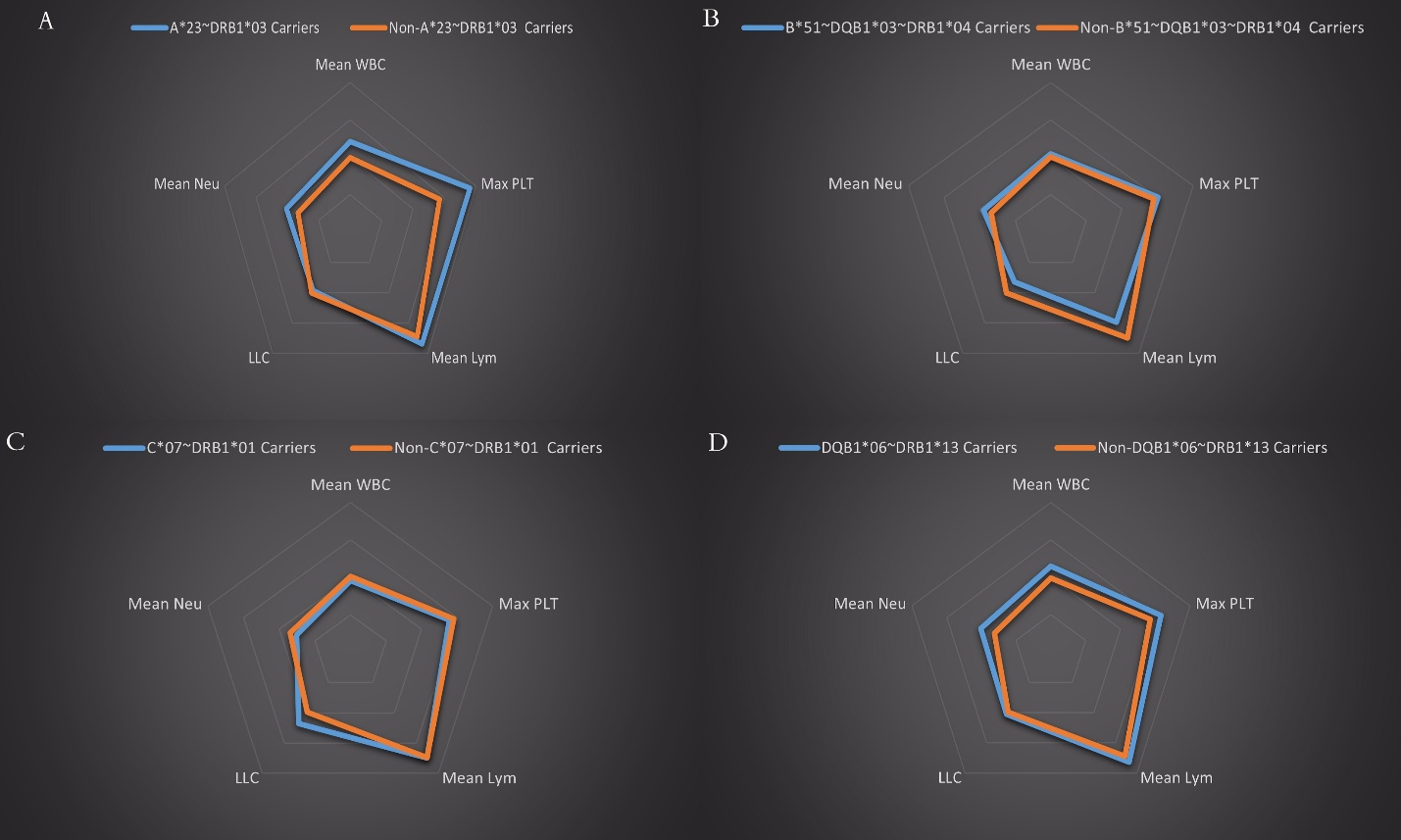


**Figure S2**. Radar chart illustrating clinically significant distinctions between individuals carrying and those not carrying specific HLA haplotypes.
